# Life-Course Pathways to Exceptional Longevity: Evidence From the Lothian Birth Cohort of 1921

**DOI:** 10.1093/gerona/glae166

**Published:** 2024-06-28

**Authors:** Janie Corley, Alison Pattie, G David Batty, Simon R Cox, Ian J Deary

**Affiliations:** Lothian Birth Cohorts, Department of Psychology, University of Edinburgh, Edinburgh, UK; Lothian Birth Cohorts, Department of Psychology, University of Edinburgh, Edinburgh, UK; Department of Epidemiology and Public Health, University College London, London, UK; Lothian Birth Cohorts, Department of Psychology, University of Edinburgh, Edinburgh, UK; Lothian Birth Cohorts, Department of Psychology, University of Edinburgh, Edinburgh, UK; (Medical Sciences Section)

**Keywords:** Health, Longitudinal, Mortality, Predictors, Survival

## Abstract

**Background:**

Longevity, a hallmark of successful aging, is a multifactorial trait with influences from birth onwards. However, limited evidence exists on the pathways linking diverse life-course exposures to longevity, especially within a single cohort.

**Methods:**

We investigated associations between life-course factors and longevity among community-dwelling adults aged 79 (*N* = 547) from the Lothian Birth Cohort 1921 with a mortality follow-up of 24 years. Cox proportional hazards and structural equation (path) models were used to explore how factors from early life (social class, childhood intelligence quotient [IQ], education), midlife (social class), and later life (health, lifestyle, psychosocial well-being), as well as sex, personality, and apolipoprotein E e4 status, influence survival time in days.

**Results:**

During follow-up (1999–2023), 538 participants (98%) died (mean age of death = 89.3 years) and 9 survived (mean age = 101.6 years). Factors associated with lower mortality risk in the multivariable Cox model were higher cognitive function (hazard ratio [HR] = 0.72; 95% confidence interval [CI]: 0.59–0.88), better physical function (HR = 0.61; 95% CI: 0.44–0.85), and greater physical activity (HR = 0.81; 95% CI: 0.71–0.92), while history of cancer was associated with higher mortality risk (HR = 1.84; 95% CI: 1.22–2.77). The life-course path model identified the same direct predictors, with additional contributions from female sex and nonsmoking status, to greater longevity. Early- and midlife factors (IQ, education, social class), and emotional stability, conscientiousness, and female sex, were indirectly and positively associated with survival trajectories via multiple dimensions of adult health.

**Conclusions:**

In understanding why people live to very old ages it is necessary to consider factors from throughout the life course, and to include demographic, psychosocial, and health variables.

As global life expectancy rises and disparities in longevity become more pronounced, understanding the diverse factors influencing individual differences in human health and longevity has become a priority research agenda for the 21st century. With advancing age comes greater vulnerability to chronic diseases, with cancer, cardiovascular diseases (CVDs), respiratory illnesses, and diabetes, being the foremost contributors to mortality among older people (1). Individuals who achieve exceptional longevity often manage to avoid, delay, or overcome these age-related conditions (2). However, longevity is a complex, multifactorial trait, with influences extending beyond comorbidities. Psychosocial factors and lifestyle behaviors such as physical inactivity, smoking, and alcohol misuse, significantly affect mortality through their associations with health and overall functioning (3,4).

Longevity, defined as living significantly longer than the average lifespan within a population, is a hallmark of successful aging. Attempts to understand longevity have largely focused on single proximal factors in adulthood that increase mortality risk, such as smoking (5), high blood pressure (6), or comorbidity such as CVD (7). A limitation of much of this research is that risk factors are treated as distinct entities and their contributions to survival are estimated in the absence of broader life-course indicators. The complexity of longevity is further compounded by distal risk factors originating from earlier life stages, which influence the aging process and pathways to disease or death (8). Early-life conditions, particularly socioeconomic disadvantages, have lasting effects on health and well-being throughout adulthood (9). Social gradients in disease, including CVD and disease-related health and lifestyle behaviors, can be traced back to childhood antecedents such as early socioeconomic adversity (10–12).

By contrast, few studies have explored the effects of early-life cognitive ability (intelligence quotient [IQ]) on survival. A systematic review concluded that early-life IQ is a robust predictor of lifespan and that childhood socioeconomic status, although correlated with childhood cognitive ability, does not fully explain its association with longevity (13). Findings across such studies are generally consistent; people with higher premorbid intelligence typically lead healthier, longer lives independently of sociodemographic risk factors (14–17). Nonetheless, relatively few studies of longevity have measured premorbid cognition (18).

A single study with life-course data can provide insights into the impact, timing, and interplay of multiple factors on longevity, examining whether childhood factors determine adult outcomes or if they are mediated, or moderated, by environmental factors and subsequent life experiences (19). Various factors can disrupt health and mortality trajectories. Education, for instance, may be a key compensatory resource, counteracting the negative trajectory initiated by early adversity (20). Behavioral factors, such as physical activity and smoking, play an intermediary role in linking early-life conditions to the biological pathways underlying chronic disease (21,22). Less well understood are attributes such as personality traits and psychological well-being and how they might be embodied to affect health and survival trajectories (23). Following a cohort from early life to death offers a potentially informative approach for exploring longevity predictors, yet few studies possess the comprehensive longitudinal characterization required to capture diverse exposures over an extended period.

Here we use data from the Lothian Birth Cohort 1921 (LBC1921), comprising individuals born over a century ago, with data at study baseline in later life at age 79, historical data from childhood and midlife periods, and mortality data collected over a 24-year period. Given survival to age 79, this study examines early-life and life-course conditions and behaviors associated with living a greater number of years beyond age 79. As such, this study represents a unique research opportunity and atypical study design that capitalizes on a deeply phenotyped cohort across many decades of life. We examine factors from early life (childhood social class, childhood IQ, education), midlife (own social class), and later life (health, lifestyle, psychosocial well-being), as well as sex, apolipoprotein E (*APOE*) e4 status, and personality. A novel aspect of our analyses involves integrating 2 distinct analytical methods: Cox proportional hazards modeling to assess the relative prognostic significance of each life-course factor for overall mortality risk, and structural equation modeling (SEM) to explore temporal (path) associations between these diverse factors from different life stages and participants’ survival.

## Method

### Participants

The LBC1921 study is a longitudinal investigation of aging comprising 550 men and women who sat an intelligence test (the Moray House Test) in childhood at age 11, as part of the Scottish Mental Survey of 1932 (24), and assessed again in later life. Baseline assessment at age 79 was conducted between 1999 and 2001 during which participants underwent comprehensive health, medical, cognitive, and other testing, and provided blood samples. The LBC1921 participants have been followed up across older age at approximately 3-year intervals, with assessments at average ages of 83, 87, 90, and 92 years. Details of the study protocol have been described elsewhere (25–27). Participants provided written informed consent. Ethics permission for the LBC1921 was obtained from the Lothian Research Ethics Committee (Wave 1: LREC/1998/4/183; Wave 2: LREC/2003/7/23; Wave 3: LREC1702/98/4/183) and the Scotland A Research Ethics Committee (Wave 4: 10/S1103/6; Wave 5: 10/MRE00/87). Inclusion criteria required mortality records or verified vital status at the time of analysis.

### Assessment of Predictor Variables

Data on potential predictor variables were collected at baseline (age 79) and categorized into early-life, midlife, later-life, and intrinsic measures, in addition to age (in days at baseline) and sex (male = 1, female = 2). All measures were continuous unless stated otherwise.

#### Early life

Early-life measures were childhood (father’s) occupational social class, age 11 IQ, and education. Father’s occupational social class was coded according to the General Register Office Census 1951 Classification of Occupations (professional–managerial [I/II], partly skilled and unskilled [III/IV/V]) (28) based on their principal occupation prior to retirement. This coincides generally with the middle portions of their careers and takes into consideration complexity of work, educational requirements, typical income, and social status. For the main prediction models, classes I/II were combined (professional/managerial = 1) and classes III/IV/V were combined (partly skilled/unskilled = 2) where a lower number denotes higher social status. Raw Moray House Test scores at age 11 were adjusted for age in days and standardized to an IQ-type score with a mean of 100 and standard deviation [*SD*] of 15, consistent with previous studies (29). Education was represented by years of full-time schooling, including further and higher education.

#### Midlife

Participants’ own occupational social class was based on principal occupation prior to retirement and coded as above (professional–managerial [I/II], partly skilled and unskilled [III/IV/V]). For the main prediction models, classes I/II were combined (professional/managerial = 1) and classes III/IV/V were combined (partly skilled/unskilled = 2) where a lower number denotes higher social status.

#### Later life

Later-life measures were from the age 79 baseline assessment and included lifestyle, physical health, physical function, cognitive function, psychosocial well-being, and emotional health. Lifestyle measures were self-reported smoking status (never = 0, former = 1, current = 2), alcohol consumption (units per week), and body mass index (BMI; kg/m^2^). A physical activity score was calculated using factor analysis of responses to all 11 items from an activity questionnaire (30) with a higher score indicating more physical activity. Health measures were self-reported history of CVD, stroke, cancer, hypertension, and diabetes (no = 0, yes = 1). Physical function was assessed by 3 directly tested measures of: forced expiratory volume in 1 second (FEV1); grip strength; and 6-m walk time (seconds). Cognitive function was measured by the Moray House Test (as above, and repeated at age 79 and converted to a standardized IQ-type score with a mean of 100 and *SD* of 15), Raven’s Matrices (31), Phonemic Verbal Fluency (32), Logical Memory (33), and National Adult Reading Test (NART); all continuous scores (34). Psychosocial well-being was measured by the Satisfaction with Life Scale (35), World Health Organization Quality of Life (36), and the Hospital Anxiety and Depression Scale (HADS) (37); all continuous scores. A higher HADS score indicates more anxiety and depressive symptoms and is referred to as emotional health in the main prediction models.

#### Intrinsic factors

In addition to age and sex, we included possession of the e4 allele of the gene for *APOE*, typed on DNA extracted from venous blood (38) on the day of assessment and included here as *APOE* e4 (no = 0, yes = 1). Two personality traits, emotional stability and conscientiousness, were measured with the 50-item International Personality Item Pool (39) and represented as continuous scores. Emotional stability is the inverse of neuroticism (the tendency toward anxiety and depression) and conscientiousness reflects self-discipline and self-control.

### Mortality Ascertainment

Dates of death were identified via data linkage with the National Health Service Central Register, provided by National Records of Scotland. Participant deaths were flagged to the research team approximately every 12 weeks beginning at study baseline in 1999. At the time of analysis, deaths certificates had been obtained for 98% of the LBC1921 sample (*N* = 538) and 9 participants (1.6%) were still alive. The remaining 3 participants were excluded from the analyses as they had relocated outwith Scotland and their status could not be verified. Our final analytical sample consisted of 547 participants, representing 99% of the original cohort. Survival data were censored at March 21, 2023, with a mean follow-up duration of 24 years. Survival time was either number of days between an individual’s birth and date of death, or number of days between birth and censor date for those still living.

### Statistical Analyses

We conducted 2 sets of analyses: one using epidemiological methods, specifically Cox proportional hazards (Cox PH) modeling with the *survival* package in R (40), and the other using psychological methods, specifically structural equation modeling (SEM) with the *lavaan* package in R (41). The Cox PH models are coded in *survival* using the format: “coxph(Surv(survival time, STATUS)).” Given that 98% of the sample were deceased, our aim was to examine survival time in days for the entire sample, including those still living, rather than comparing those alive versus deceased. As such, we used the following coding: “coxph(Surv(survival in days, DEAD))” where survival in days was calculated as the number of days between an individual’s birth date and their date of death (*N* = 538) or the number of days between birth date and right-censored date at time of analysis (March 21, 2023) for those still living (*N* = 9). In doing so, all participants were treated equally with the outcome being how long they survived in days rather than whether or not they died by the study end point.

First, we ran a series of individual univariate Cox PH models to estimate the effect of each predictor variable on mortality risk, controlling for age and sex. Then, we ran a single multivariable Cox PH model to estimate the relative effect of each predictor on mortality risk while simultaneously adjusting for all other variables. Hazard ratios (HRs) and 95% confidence intervals (CIs) are presented. Model performance of the multivariable model was assessed using the Concordance Index (where higher values indicate better prediction performance) and the Likelihood Ratio test assessed goodness of fit.

Next, SEM techniques were used to examine the temporal relationships between measures at different life stages and their associations with survival time. Direct paths were specified from each measure to survival. Additionally, in accordance with the life-course approach, paths were also specified from the early- and midlife measures (childhood occupational social class, age 11 IQ, education, midlife occupational social class) to the later-life measures (CVD, stroke, cancer, hypertension, diabetes, smoking, activity, alcohol, BMI, life satisfaction, quality of life, emotional health, physical function, and cognitive function). Further paths were specified from age, sex, emotional stability, and conscientiousness (ie, “intrinsic” factors) to the later-life measures. For the direct paths in the structural model, HRs were calculated as exponentiated beta coefficients; therefore, 95% CIs for these HRs were unable to be determined. Effect sizes for these and all other paths are represented by standardized beta (std β) coefficients.

Prior to analysis, continuous measures were *z*-scored, and latent factors were constructed for physical function and cognitive function, due to high correlations among the individual direct measurements within each domain. The latent factors were calculated using confirmatory factor analysis within the *lavaan* package. The latent factor of physical function, explaining 46% of the variance, was derived from 3 direct measures of FEV1 (std β loading = 0.76), grip strength (std β loading = 0.84), and 6-m walk scores (std β loading = −0.308) and adjusted for height. The latent measure of cognitive function, explaining 48% of the variance, was derived from 5 cognitive test scores at age 79, namely the Moray House Test (std β loading = 0.96), Raven’s Matrices (std β loading = 0.74), Logical Memory (std β loading = 0.47), Verbal Fluency (std β loading = 0.45), and NART (std β loading = 0.70). The standardized latent factor scores (mean = 0) were used as continuous variables, with higher scores indicating better physical or cognitive function, respectively.

#### SEM fit and significance statistics

The structural model was run using full information maximum likelihood (FIML) estimation to reduce bias due to missing data. Model fit was tested using the root mean square error of approximation (RMSEA) and the standardized root mean square residual (SRMR) with values of ≤0.08 for the RMSEA and ≤0.06 for the SRMR indicating good model fit. Correction for multiple comparisons was applied across both sets of prediction models using the false discovery rate (FDR) (42) adjustment, and results marked in bold type are those that survive FDR correction.

#### Sensitivity analysis

In response to reviewer feedback, we reran the same multivariable Cox PH model by right-censoring at an earlier planned time point when there were more survivors, to test our hypothesis that the same pattern of results apparent for predictors in the current analyses would be replicated in analyses based on right-censoring 10 years prior (March 21, 2013) to the original census date.

## Results

Table 1 presents the baseline characteristics of the LBC1921 of which 57.5% were female. Approximately one-third had a childhood (father’s) social class of I or II, indicative of higher job status, and around half had an own (midlife) social class of I or II. Just over a half reported a history of smoking. The average survival age across the analytic sample (*N* = 547) was 89.5 years (*SD* = 5.3), including participants who had died (*N* = 538, mean age at death = 89.3 years, *SD* = 5.1) and those participants alive at the end of follow-up (*N* = 9, mean age at census date = 101.6 years, *SD* = 0.5). Supplementary Table 1 presents a correlation matrix of all model variables. Supplementary Table 2 presents variable information and missing data at baseline, and Supplementary Table 3 presents participant characteristics based on data completeness. Participants with incomplete data on at least 1 covariate (*N* = 206) had significantly lower age 11 and age 79 cognitive scores, more smoking and a higher alcohol intake, lower physical activity levels, slower 6-m walk times, lower quality of life, and a greater likelihood of diabetes and hypertension. Note that the main prediction models used FIML estimation to mitigate bias due to missingness.

**Table 1.** Baseline Characteristics of the Lothian Birth Cohort 1921

	Full Sample	Men	Women
Age (years)	79.07 (0.6)	79.08 (0.6)	79.07 (0.6)
Survival time (years)	89.50 (5.3)	88.90 (5.3)	89.94 (5.3)
Early life			
Childhood social class, *N* (%)			
I (professional)	47 (10.0)	24 (12.0)	23 (8.7)
II	123 (26.3)	52 (26.0)	71 (26.5)
III	228 (48.7)	100 (50.0)	128 (47.8)
IV	50 (10.7)	16 (8.0)	34 (12.7)
V (unskilled)	20 (4.3)	8 (4.0)	12 (4.5)
Age 11 IQ score	100.00 (15.0)	99.49 (15.5)	100.37 (14.6)
Education (years)	10.92 (2.5)	11.26 (2.8)	10.67 (2.2)
Midlife			
Midlife social class, *N* (%)			
I (professional)	129 (23.5)	67 (28.6)	62 (19.7)
II	183 (33.4)	77 (32.9)	106 (33.8)
III	217 (39.6)	84 (35.9)	133 (42.4)
IV	12 (2.2)	3 (1.3)	9 (2.9)
V (unskilled)	7 (1.3)	3 (1.3)	4 (1.3)
Later life			
Smoking, *N* (%)			
Never	238 (43.4)	81 (34.6)	157 (49.8)
Former	271 (49.4)	138 (59.0)	133 (42.2)
Current	40 (7.3)	15 (6.4)	25 (7.9)
Cardiovascular disease, *N* (%)	163 (29.6)	90 (38.8)	73 (23.3)
Stroke, *N* (%)	46 (8.4)	24 (10.3)	22 (7.0)
Cancer, *N* (%)	54 (9.8)	27 (11.5)	27 (8.5)
Hypertension, *N* (%)	220 (40.0)	86 (36.9)	134 (42.5)
Diabetes, *N* (%)	28 (5.1)	13 (5.6)	15 (4.7)
*APOE* e4 carrier, *N* (%)	145 (26.7)	57 (25.0)	88 (38.8)
Alcohol intake (units per week)	5.77 (10.7)	8.80 (14.0)	3.52 (6.5)
Physical activity score	22.30 (5.7)	21.68 (5.5)	22.77 (4.6)
Body mass index	26.23 (4.2)	26.23 (3.5)	26.23 (4.6)
Forced expiratory volume in 1 second	1.88 (0.6)	2.34 (0.6)	1.55 (0.4)
Grip strength	26.54 (9.1)	34.71 (7.4)	20.60 (4.5)
Six-meter walk (seconds)^a^	4.74 (1.9)	4.38 (1.7)	5.00 (2.1)
Age 79 IQ score	100 (15.0)	101.82 (14.7)	98.68 (15.1)
Raven’s Matrices score	31.16 (8.8)	32.55 (8.7)	30.14 (8.7)
Verbal Fluency score	40.00 (12.3)	39.55 (12.6)	40.33 (12.2)
Logical Memory score	31.64 (12.8)	32.25 (13.2)	31.20 (12.5)
National Adult Reading Test score	34.16 (8.3)	34.17 (8.4)	34.15 (8.3)
Emotional health (HADS)	8.73 (4.7)	8.19 (4.6)	9.13 (4.8)
Quality of Life (WHOQOL)	63.91 (7.3)	64.61 (7.1)	63.37 (7.5)
Satisfaction with life (SWLS)	25.34 (6.1)	25.72 (6.4)	25.05 (5.8)
Emotional stability	24.34 (8.1)	24.86 (8.5)	23.98 (7.9)
Conscientiousness	28.71 (6.1)	28.62 (6.0)	28.77 (6.1)

*Notes*: *APOE* = apolipoprotein E; HADS = Hospital Anxiety and Depression Scale; IQ = intelligence quotient; SWLS = Satisfaction with Life Scale; WHOQOL = World Health Organization Quality of Life. Unless otherwise stated, results are mean (standard deviation).

^a^A higher value denotes slower walking speed.

### Cox Proportional Hazards Models

#### Univariable models

Table 2 (left columns) presents the univariable Cox PH models, showing associations between each predictor variable, alongside age and sex, and mortality risk. Several predictors were significantly associated with lower risk of mortality after FDR correction: higher physical activity (HR = 0.78, CI = 0.71, 0.86); higher cognitive function (HR = 0.79, CI = 0.72, 0.87); greater physical function (HR = 0.50, CI = 0.40, 0.64); and higher quality of life (HR = 0.85, CI = 0.77, 0.93). Female sex and more education were also associated with living longer but these associations were smaller and not robust to FDR correction. Predictors significantly associated with higher mortality risk in the univariable model were history of cancer (HR = 1.98, CI = 1.49, 2.64), current smoking (HR = 1.55, CI = 1.10, 2.17, compared with never smoking), and diabetes (HR = 1.89, CI = 1.29, 2.77). Additionally, lower childhood social class, former smoking (compared with never smoking), *APOE* e4, history of hypertension, and poorer emotional health were associated with lower survival, but these associations were smaller and not robust to FDR correction.

**Table 2.** Cox Proportional Hazards Models Predicting Mortality

	Univariable Models (Age, Sex + Each Covariate)^a^		Multivariable Model (All Covariates Mutually Adjusted)^b^	
	Hazard Ratios (95% CI)	*p* Value	Hazard Ratios (95% CI)	*p* Value
Age (in days)	0.99 (0.91, 1.08)	.853	0.92 (0.82, 1.03)	.170
Sex (ref: males)	0.83 (0.70, 0.99)	.033^c^	0.86 (0.66, 1.12)	.269
Early life				
Social class (ref: prof/managerial)				
Partly skilled/unskilled	1.21 (1.00, 1.48)	.042^c^	1.28 (0.99, 1.65)	.055
Age 11 IQ score	0.94 (0.87, 1.04)	.258	1.13 (0.96, 1.33)	.156
Education (years)	0.96 (0.93, 0.99)	.019^c^	0.98 (0.93, 1.04)	.578
Midlife				
Social class (ref: prof/managerial)				
Partly skilled/unskilled	1.18 (0.99, 1.30)	.062	0.95 (0.74, 1.23)	.693
Later life: lifestyle				
Smoking (ref: never)				
Former smoking	1.23 (1.03, 1.47)	.022^c^	1.08 (0.85, 1.38)	.515
Current smoking	1.55 (1.10, 2.17)	**.011**	1.24 (0.74, 2.09)	.419
Alcohol intake (units)	1.03 (0.94, 1.14)	.505	1.16 (0.99, 1.36)	.063
Physical activity score	0.78 (0.71, 0.86)	**<.001**	0.81 (0.71, 0.92)	**.001**
Body mass index	1.04 (0.95, 1.12)	.416	0.98 (0.87, 1.10)	.695
Later life: physical health				
CVD (ref: no history)	1.19 (0.98, 1.42)	.085	1.13 (0.87, 1.47)	.363
Stroke (ref: no history)	0.97 (0.72, 1.32)	.865	0.88 (0.60, 1.29)	.521
Cancer (ref: no history)	1.98 (1.49, 2.64)	**<.001**	1.84 (1.22, 2.77)	**.004**
Hypertension (ref: no history)	1.19 (1.00, 1.42)	.045^c^	1.19 (0.94, 1.51)	.144
Diabetes (ref: no history)	1.89 (1.29, 2.77)	**.001**	1.90 (1.05, 3.46)	.035^c^
Later life: functional health				
Physical function (latent factor)	0.50 (0.40, 0.64)	**<.001**	0.61 (0.44, 0.85)	**.003**
FEV in 1 second	0.78 (0.72, 0.86)	**<.001**		
Grip strength	0.85 (0.77, 0.93)	**<.001**		
Six-meter walk (seconds)	1.29 (1.18, 1.41)	**<.001**		
Later life: cognitive health				
Cognitive function (latent factor)	0.79 (0.72, 0.87)	**<.001**	0.72 (0.59, 0.88)	**.001**
Age 79 IQ score	0.82 (0.75, 0.89)	**<.001**		
Raven’s Matrices score	0.82 (0.75, 0.89)	**<.001**		
Verbal Fluency score	0.89 (0.82, 0.97)	**.009**		
Logical Memory score	0.86 (0.79, 0.93)	**<.001**		
NART score	0.89 (0.82, 0.97)	**.006**		
Later life: psychosocial health				
Emotional health (HADS)	1.10 (1.00, 1.20)	.040^c^	0.94 (0.82, 1.08)	.410
Quality of life (WHOQOL)	0.85 (0.77, 0.93)	**<.001**	1.05 (0.87, 1.28)	.587
Satisfaction with life (SWLS)	0.96 (0.87, 1.05)	.342	1.04 (0.90, 1.20)	.637
Intrinsic: genetic				
* APOE* e4 status (ref: −ve)	1.23 (1.01, 1.49)	.038^c^	0.94 (0.72, 1.22)	.642
Intrinsic: personality				
Emotional stability	0.92 (0.83, 1.01)	.078	0.93 (0.81, 1.07)	.332
Conscientiousness	0.97 (0.89, 1.07)	.568	0.99 (0.88, 1.12)	.910

*Notes*: *APOE* = apolipoprotein E; CI = confidence interval; CVD = cardiovascular disease; FEV = forced expiratory volume; FDR = false discovery rate; FEV1, forced expiratory volume in 1 second; HADS = Hospital Anxiety and Depression Scale; IQ = intelligence quotient; SWLS = Satisfaction with Life Scale; WHOQOL = World Health Organization Quality of Life. Survival time was calculated as either the number of days between date of birth and date of death or right census date. Unless indicated with a corresponding reference category, measures were continuous. Bold type indicates *p* values that survive FDR adjustment.

^a^For the univariable models, each individual measure was examined in a separate model (alongside age and sex). Additionally, we examined univariable associations of a latent measure of physical function and a latent measure of cognitive function.

^b^For the multivariable model, we examined all measures simultaneously in 1 single model. Here, the latent measures of physical function and cognitive function were entered instead of the individual measures from which they were derived.

^c^Indicates statistical significance at the *p* < .05 level, before FDR correction.

#### Multivariable model

**Figure 1.**
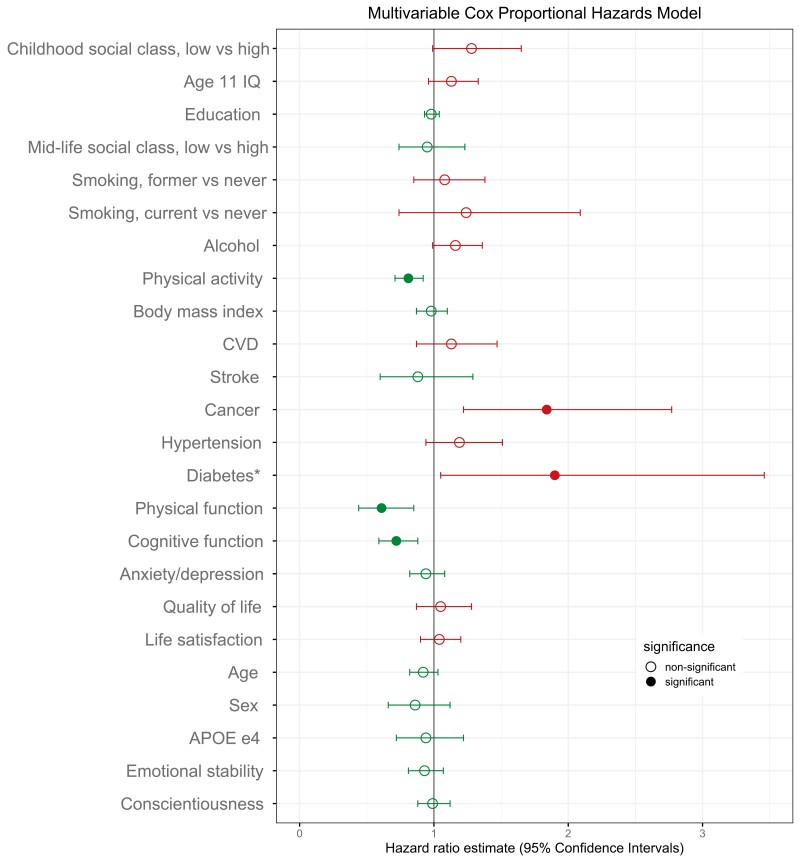
Multivariable-adjusted Cox proportional hazard ratios of each predictor for mortality risk. Corresponding estimates are shown in Table 2 (right columns). Values in green denote a lower mortality risk, values in red denote a higher mortality risk. *Non-FDR significant. *APOE* = apolipoprotein E; CVD = cardiovascular disease; FDR = false discovery rate; IQ = intelligence quotient.

Table 2 (right columns) presents the results from the multivariable “all-in” model, which collectively examined the associations of all predictor variables with survival to determine which predictors remained significant after mutual adjustment. Three predictors maintained FDR-significant associations with lower mortality risk (ie, greater survival): greater physical activity (HR = 0.81, CI = 0.71, 0.92); higher cognitive function (HR = 0.72, CI = 0.59, 0.88); and higher physical function (HR = 0.61, CI = 0.44, 0.85), while history of cancer was associated with a higher mortality risk (HR = 1.84, CI = 1.22, 2.77). A smaller association between diabetes and mortality risk was nonsignificant after FDR correction. The Concordance Index for this multivariable model was 0.63 (*p* < .0001) indicating good prediction performance and the Likelihood Ratio of 71.70 (*p* < .0001) indicated a significant improvement in model fit compared to a null model (ie, a model with no predictors). Figure 1 is a visual representation of these multivariable results in a forest plot.

### Sensitivity Analysis

The sensitivity analysis (Supplementary Table 4) using an earlier census date 10 years prior to the original, when there were more survivors (*N* = 199 alive, *N* = 348 dead), confirmed that the FDR-significant predictors of longevity—physical activity, physical function, cognitive function, and cancer history—remained consistent, in the same direction. Smaller associations were observed between lower survival and a history of diabetes, higher alcohol consumption, and lower childhood social class, which were significant but not robust to FDR correction.

### Structural (Life-Course Path) Model

**Figure 2.**
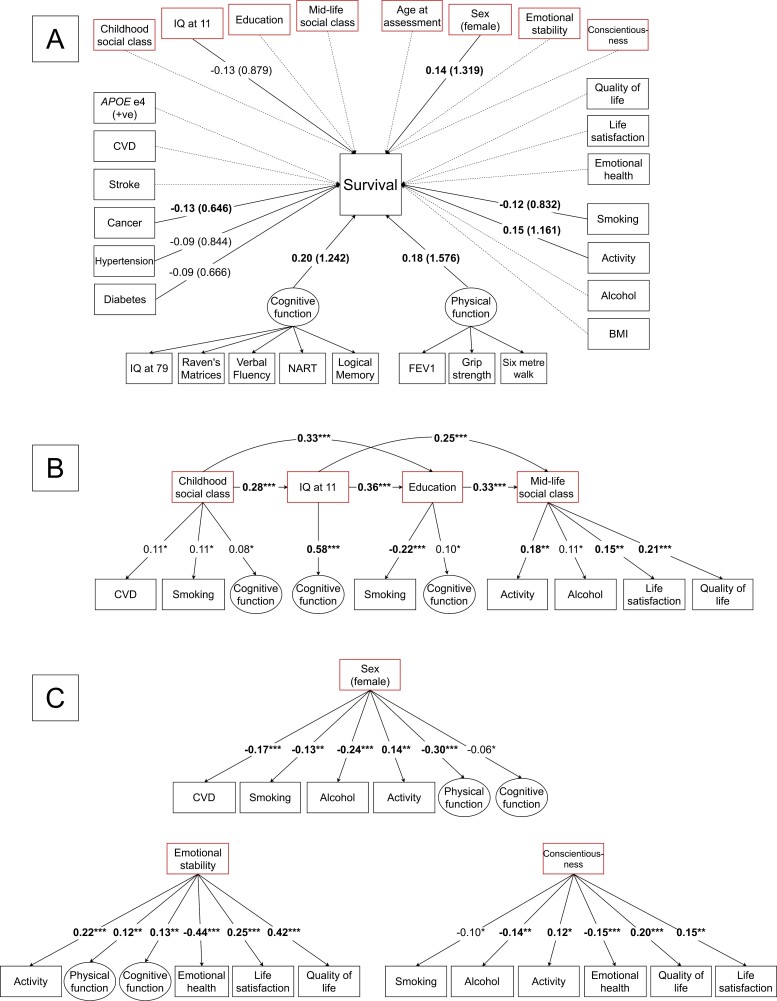
Structural (path) model of associations between predictors and survival time. Parts A, B, and C are derived from a single SEM model. A shows the predictors’ direct effects on survival. Values are std beta coefficients and values in parentheses are exponentiated path coefficients (hazard ratios). Bold type indicates FDR-significant associations. Dashed lines indicate nonsignificant paths. Red boxes indicate variables with additional specified paths to later-life variables as shown in B and C. B shows significant temporal paths from the earlier life-course variables (childhood social class, IQ at 11, education, midlife social class) and the age 79 variables. C shows the significant paths from the intrinsic factors (age, sex, and personality traits) to the later-life variables. For B and C, values shown are std beta coefficients (*p < .05, **p < .01, ***p < .001) and bold type indicates FDR-significant associations. Only significant paths are presented to reduce visual clutter; full results are presented in Supplementary Table 5. *APOE* = apolipoprotein E; CVD = cardiovascular disease; FDR = false discovery rate; FEV1 = forced expiratory volume in 1 second; IQ = intelligence quotient; NART = National Adult Reading Test; SEM = structural equation modeling; std = standardized.

Using SEM, we investigated direct associations between all model variables and survival time (in days), incorporating additional pathways to examine temporal associations between factors across different life stages. The model demonstrated acceptable fit based on SRMR (0.05) and RMSEA (0.08) fit indices. Figure 2 presents the structural model as a path diagram divided into 3 parts for visual clarity: significant direct effects on survival (A); temporal paths from earlier- to later-life variables (B); and intrinsic factors to later-life variables (C). Full estimates are provided in Supplementary Table 5.

#### Direct paths to survival

**Figure 3.**
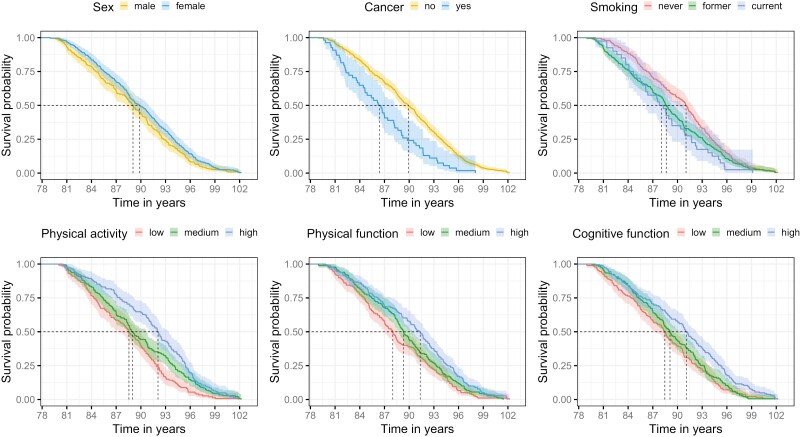
Survival curves for the 6 predictors with FDR-significant direct effects on survival in the structural (path) model (see Supplementary Table 5). Survival is represented here in years for clarity but modeled as survival in days. The variables for physical activity, physical function, and cognitive function were split into tertiles for visual illustration only; in the models, these were treated as continuous variables. The black dashed lines represent the median survival age for each category. FDR = false discovery rate.

The key predictors of survival with FDR-significant direct effects ordered from highest to lowest effect size (std β) were age 79 cognitive function (β = 0.20, *p* < .001; HR = 1.24), physical function (β = 0.18, *p* < .001; HR = 1.58), physical activity (β = 0.15, *p* = .002; HR = 1.16), sex (β = 0.14, *p* = .002, HR = 1.14), and history of cancer (β = −0.13, *p* = .001; HR = 0.65), and smoking (β = −0.12, *p* = .005; HR = 0.83). Positive beta coefficients indicate a positive effect on survival and negative coefficients indicate an adverse effect. Figure 3 displays survival curves for these 6 variables. Additional direct associations with survival, not robust to FDR adjustment, included diabetes (β = −0.09, *p* = .025; HR = 0.67), hypertension (β = −0.09, *p* = .038; HR = 0.84), and IQ at age 11 (β = −0.13, *p* = .036; HR = 0.88).

#### Temporal paths between life-course factors

The earlier-life variables (childhood social class, age 11 IQ, education, own midlife social class) demonstrated downstream associations with at least 1 later-life variable (see Figure 2B) though not all survived FDR adjustment. The most robust associations included: age 11 IQ with later-life cognitive function (β = 0.58, *p* < .001); higher (more professional) own social class with greater physical activity (β = 0.18, *p* < .001), life satisfaction (β = 0.15, *p* = .004), and quality of life (β = 0.21, *p* < .001); and more education with less smoking (β = −0.22, *p* < .001). Associations from childhood social class to later-life variables were marginally (non-FDR) significant, namely that a more professional social class of origin was associated with higher cognitive function (β = 0.08, *p* = .043), more CVD (β = 0.11, *p* = .034), and more smoking (β = 0.11, *p* = .038).

Of the “intrinsic” factors, age was not associated with any of the later-life variables. However, there were numerous significant paths from sex, emotional stability, and conscientiousness to health and well-being measures in later life (see Figure 2C). For sex, women showed less smoking (β = −0.13, *p* = .002) and alcohol consumption (β = −0.24, *p* < .001), more physical activity (β = 0.14, *p* = .001), and lower rates of CVD (β = −0.17, *p* < .001), but poorer physical function (β = −0.30, *p* < .001) and marginally poorer cognitive function (β = −0.06, *p* = .043, non-FDR significant). Higher emotional stability was associated with more positive outcomes including more physical activity (β = 0.22, *p* < .001), higher physical function (β = 0.12, *p* = .008), higher cognitive function (β = 0.13, *p* < .001), better emotional health (ie, lower anxiety and depression; β = −0.44, *p* < .001), better quality of life (β = 0.42, *p* < .001), and life satisfaction (β = 0.25, *p* < .001). Higher conscientiousness was also associated with positive outcomes including better emotional health (ie, lower anxiety and depression; β = −0.15, *p* < .001), better quality of life (β = 0.20, *p* < .001), life satisfaction (β = 0.15, *p* = 0.002), more physical activity (β = 0.12, *p* = .001), less alcohol consumption (β = −0.14, *p* = .005), and marginally lower rates of smoking (β = −0.10, *p* = .043, non-FDR significant).

## Discussion

Our principal finding, shown by the multivariable Cox model, indicated that low physical activity, low cognitive function and physical function, and a history of cancer, were the main factors associated with higher mortality risk in this older cohort. The structural equation model provided a more nuanced examination of the temporal order of life-course determinants, showing direct and indirect associations between lifetime exposures and health and survival in advanced age. Longer-lived individuals were more likely to be women, those with higher cognitive and physical functions and greater physical activity at age 79, and lower rates of lifetime smoking and cancer. Cognitive ability emerged as the strongest unique predictor of longevity, with a moderate effect size (std β = 0.20), surpassing physical function.

Earlier-life factors—childhood social class, childhood IQ, education, and midlife social class—alongside emotional stability and conscientiousness, also contributed to longevity via positive downstream associations with later-life health, lifestyle, and well-being. These results suggest that favorable childhood conditions and positive personality traits foster resilient pathways, shaping survival trajectories. Our study provides novel evidence of unique and shared pathways linking specific life-course factors from different periods to health and longevity, which are not always apparent in traditional (epidemiologic) survival analyses or cross-sectional studies.

Our study confirms the relationship between common risk factors for mortality such as male gender, chronic disease, low physical function, and low physical activity (43), and extends the literature by suggesting that: (i) later-life cognitive ability is an important proximal predictor of survival in the oldest old, even when accounting for common risk factors; (ii) childhood conditions, such as education and early social class, continue to affect physical health up to 70 years later; (iii) higher emotional stability and conscientiousness may indirectly confer a longevity advantage via a cascade of positive effects on physical, psychological, and psychosocial health and well-being domains.

### Comparisons With Existing Studies

Previous studies have reported associations between life-course factors and longevity (44,45) but often with shorter follow-ups and younger samples, meaning their results may not have fully captured the predictors of longevity in those exceptionally long-lived as well as their slightly younger peers. For example, the Health and Retirement study (13 611 men and women, aged 52–104, mean age 69) found childhood socioeconomic adversity inversely associated with longevity after 6 years of follow-up, alongside common risk factors for mortality such as smoking, alcohol abuse, lack of physical activity, and negative affect (44). The Manitoba Follow-up Study (3 976 men, followed from 1948 until their 90th birthday) found that predictors of survival shift across the life course: childhood illness was important in early life, chronic illness in midlife, and quality of life in later life (45). Crucially, these studies modeled potential predictors from various life stages simultaneously, but not temporally.

Two previous studies explored life-course pathways to longevity. In the Terman Life Cycle study, childhood conscientiousness predicted better family relationships, and longer life, in 1 021 participants living to ≥85 years (46). Among 1 042 men from the Normative Aging study aged ~60 at baseline, stressful life experiences were found to compound the negative influence of childhood psychosocial adversity on reduced longevity, while midlife optimism and life satisfaction led to more positive trajectories (47). While these studies identified pathways linking life-course factors and survival, both focused on psychosocial measures, and did not examine common risk factors such as chronic disease and lifestyle, nor did they explore how childhood cognitive ability relates to health and how long an individual survives.

Higher cognitive function at age 79 emerged as the strongest predictor of survival in the life-course model with all 3 early-life factors (childhood social class, age 11 IQ, education) contributing to this outcome. This supports evidence from the Danish Birth Cohort Studies, which found that cognitive performance on the Mini-Mental State Examination was a good predictor of centenarian survival (48). Our study extends these findings to a more comprehensive measure of cognitive function using tests that are sensitive to subtle, age-related cognitive changes in nonclinical groups. Terminal decline—the drop in mental functioning shortly before death—may explain the observed association between lower cognitive ability at 79 and reduced longevity (49), although cognitive function at age 79 remained a significant predictor of mortality even when an earlier census date by 10 years was applied in a sensitivity analysis. However, the association between lower cognitive function and higher mortality risk is complex and does not imply direct causation. Poor cognitive function is often a marker for underlying health conditions, such as neurodegenerative diseases, cardiovascular issues, and frailty (50–52), which are on the causal pathway to mortality. While previous studies suggested a protective effect of childhood intelligence on longevity (13,14), we could not confirm this. Our structural model showed an unexpected inverse association between childhood IQ and survival, possibly due to statistical complexities arising from multiple covariates with overlapping pathways. This result is likely to reflect the residual variance accounted for by age 11 IQ, which is not already shared via the other covariates. This association was marginal and became nonsignificant after FDR correction. Moreover, it is important to note that childhood IQ had a positive albeit nonsignificant association with survival time in the univariable and multivariable Cox PH models.

We conducted conventional epidemiologic analyses (ie, Cox PH) alongside conventional psychological analyses (ie, SEM). These analyses are complementary and, in this case, confirmatory; most significant predictors were consistent across both multivariable models. Minor differences between the Cox and SEM models can be attributed to their handling of temporal factors and missing data. The structural model’s use of FIML mitigated missing data issues, enhancing robustness, whereas the Cox PH model may be biased by missing data patterns.

The path model demonstrates that earlier-life factors continue to influence longevity, even if not directly, many decades later. Late-life health outcomes including longevity are, to a considerable extent, influenced by conditions earlier and even very early in life, including childhood. Lower childhood and adult social class and less education were indirectly associated with shorter lifespan through unhealthy lifestyles (smoking, higher alcohol intake, low physical activity), chronic disease (such as CVD), and reduced life satisfaction. This study underscores the value of a life-course approach in identifying vulnerable populations at risk of ill-health and increased mortality. For example, the adverse direct effects of smoking on survival could be traced back to early-life disadvantage, particularly lower education levels. One of the useful extensions to the present study would be to inquire which personal attributes and behaviors are associated with long life in those individuals who had less favorable early-life experiences and environments.

### Strengths and Limitations

Strengths of the study include comprehensive life-course data in a single cohort, near-complete mortality ascertainment over 24 years in a long-lived sample, and a life-course approach to modeling predictors of longevity. The cohort’s exceptional longevity (averaging 89.5 years) exceeds the average age of survival in Scotland (78.6 years) by over 10 years, and markedly surpasses life expectancy for their birth year of 1921 (53.1 years for males and 56.4 years for females) in Scotland (53) by at least 3 decades, indicating remarkable achievement. As such, we have addressed 3 criticisms often leveled at studies of mortality prediction: (i) an inability to examine diverse factors from different fields of research simultaneously; (ii) a lack of early-life data; and (iii) relatively short follow-up periods of “younger” samples. Our study modeled social class and cognitive ability across the life course, combined with genetic, lifestyle, personality, psychosocial, and health data, providing a well-rounded view of health and functioning and robust estimates of each exposure’s relative contributions to subsequent longevity. Using FIML minimized the impact of missing covariate data and bias against those with incomplete information.

Despite these strengths, our study has limitations. The relatively small, healthy volunteer sample introduces survivor bias, limiting the generalizability of our findings. Reaching octogenarian status and beyond is proof of successful aging; less healthy individuals may have died before the age of 79 years when study recruitment commenced. This bias likely results in weaker associations between measures and longevity than actually exist. Reliance on self-reports of disease may have resulted in underestimates of disease prevalence. Predictors were largely captured in later life with limited midlife data (occupational social class only). We did not examine pathways between later-life predictors, for example, the effect of greater physical activity on longevity may have acted through lowering cardiac risk factors. Finally, we modeled predictors at baseline rather than over time. Notwithstanding additional levels of complexity within the models, determining the prognostic significance of baseline predictors, that is—what can information about individuals at study recruitment tells us about their future mortality risk—has inherent value within the context of longitudinal studies.

## Conclusion

Beyond common risk factors, such as gender, chronic disease, and poor lifestyle, our study highlights the contributions of cognitive ability in later life to longevity, and the impact of earlier-life experiences and personality traits on health and survival. By adopting a temporal, life-course perspective within a long-lived cohort, our findings offer a nuanced understanding of determinants of survival to older old age.

## Supplementary Material

glae166_suppl_Supplementary_Materials

## Data Availability

Data from this study can be requested from the Lothian Birth Cohorts. Further information, including data request form, data summaries, and data dictionaries, can be found here: https://www.ed.ac.uk/lothian-birth-cohorts.
