# Supplementary material for: Life-Course Pathways to Exceptional Longevity: Evidence From the Lothian Birth Cohort of 1921
Source: J Gerontol A Biol Sci Med Sci. 2024 Jun 28;79(8):glae166. doi: 10.1093/gerona/glae166 (PMC11285160; doi:10.1093/gerona/glae166)
Supplement: glae166_suppl_Supplementary_Materials [file glae166_suppl_supplementary_materials.docx]

**Supplementary Material**

Table of Contents

**eTable 1.** Correlation matrix of associations between all predictors page 2

**eTable 2.** Variable information and missing data at baseline page 3

**eTable 3.** Characteristics of the sample according to full vs incomplete data page 4

**eTable 4**. Sensitivity analysis: Multivariable Cox proportional hazards model predicting mortality with an

earlier census date (21 March 2013) page 6

**eTable 5.** Structural life-course model predicting survival: includes direct paths to survival from all covariates, and

additional paths among early-life variables, and from early-life and intrinsic (stable) variables to later-life variables page 8

**eTable 1.** Correlation matrix of associations between all predictors

|  | 1 | 2 | 3 | 4 | 5 | 6 | 7 | 8 | 9 | 10 | 11 | 12 | 13 | 14 | 15 | 16 | 17 | 18 | 19 | 20 | 21 | 22 | 23 | 24 | 25 | 26 | 27 | 28 | 29 |
| --- | --- | --- | --- | --- | --- | --- | --- | --- | --- | --- | --- | --- | --- | --- | --- | --- | --- | --- | --- | --- | --- | --- | --- | --- | --- | --- | --- | --- | --- |
| 1 Age | 1 |  |  |  |  |  |  |  |  |  |  |  |  |  |  |  |  |  |  |  |  |  |  |  |  |  |  |  |  |
| 2 Sex | -.018 | 1 |  |  |  |  |  |  |  |  |  |  |  |  |  |  |  |  |  |  |  |  |  |  |  |  |  |  |  |
| 3 Age11 IQ | -.031 | .035 | 1 |  |  |  |  |  |  |  |  |  |  |  |  |  |  |  |  |  |  |  |  |  |  |  |  |  |  |
| 4 Childhood SES | .031 | .061 | **.260** | 1 |  |  |  |  |  |  |  |  |  |  |  |  |  |  |  |  |  |  |  |  |  |  |  |  |  |
| 5 Education | -.018 | **-.113** | **.431** | **.416** | 1 |  |  |  |  |  |  |  |  |  |  |  |  |  |  |  |  |  |  |  |  |  |  |  |  |
| 6 Mid-life SES | .067 | **-.101** | **.409** | **.287** | **.478** | 1 |  |  |  |  |  |  |  |  |  |  |  |  |  |  |  |  |  |  |  |  |  |  |  |
| 7 Smoking | -.011 | **-.114** | -.058 | -.026 | **-.144** | -.036 | 1 |  |  |  |  |  |  |  |  |  |  |  |  |  |  |  |  |  |  |  |  |  |  |
| 8 Alcohol | .004 | **-.249** | .037 | **-.111** | **.096** | **.153** | **.136** | 1 |  |  |  |  |  |  |  |  |  |  |  |  |  |  |  |  |  |  |  |  |  |
| 9 Physical activity | -.017 | **.095** | .083 | **.125** | **.168** | **.196** | **-.141** | -.012 | 1 |  |  |  |  |  |  |  |  |  |  |  |  |  |  |  |  |  |  |  |  |
| 10 Body mass index | .004 | .004 | -.050 | .047 | -.035 | -.066 | -.019 | -.008 | -.085 | 1 |  |  |  |  |  |  |  |  |  |  |  |  |  |  |  |  |  |  |  |
| 11 CVD | .034 | **-.170** | -.048 | -.077 | -.036 | -.022 | .033 | .007 | **-.112** | -.001 | 1 |  |  |  |  |  |  |  |  |  |  |  |  |  |  |  |  |  |  |
| 12 Stroke | -.008 | -.060 | .006 | .042 | -.010 | .010 | -.004 | -.036 | .002 | -.035 | .**138** | 1 |  |  |  |  |  |  |  |  |  |  |  |  |  |  |  |  |  |
| 13 Cancer | -.041 | -.051 | .009 | .012 | -.021 | -.019 | .061 | .029 | -.026 | -.035 | .024 | .032 | 1 |  |  |  |  |  |  |  |  |  |  |  |  |  |  |  |  |
| 14 Hypertension | .007 | .059 | -.004 | .037 | -.044 | .003 | -.041 | -.045 | -.037 | **.163** | .051 | **.142** | -.080 | 1 |  |  |  |  |  |  |  |  |  |  |  |  |  |  |  |
| 15 Diabetes | .029 | -.019 | .018 | -.024 | .008 | -.011 | .015 | -.030 | -.085 | **.157** | **.109** | .079 | .062 | .064 | 1 |  |  |  |  |  |  |  |  |  |  |  |  |  |  |
| 16 *APOE* e4 | -.031 | .031 | -.053 | .006 | **-.097** | -.021 | .039 | **-.088** | -.051 | -.028 | .040 | .025 | **.086** | .030 | -.066 | 1 |  |  |  |  |  |  |  |  |  |  |  |  |  |
| 17 FEV in 1 sec | -.047 | **-.617** | **.098** | **.150** | **.282** | **.216** | -.048 | **.159** | .064 | **-.093** | .075 | .002 | -.044 | **-.089** | -.005 | -.029 | 1 |  |  |  |  |  |  |  |  |  |  |  |  |
| 18 Grip strength | -.045 | **-.764** | .027 | -.075 | **.175** | **.134** | **.104** | **.145** | -.043 | -.009 | **.095** | .012 | -.016 | -.046 | -.017 | -.015 | **.644** | 1 |  |  |  |  |  |  |  |  |  |  |  |
| 19 Six metre walk | .071 | **.155** | .013 | **-.145** | **-.093** | **-.106** | .055 | .023 | **-.319** | **.252** | **.123** | .015 | .001 | .012 | **.265** | .002 | **-.235** | **-.259** | 1 |  |  |  |  |  |  |  |  |  |  |
| 20 Age79 IQ | -.007 | **-.099** | **.656** | **.292** | **.419** | **.365** | -.057 | .033 | **.157** | -.061 | -.033 | -.042 | -.046 | .026 | -.030 | **-.114** | **.229** | **.191** | **-.215** | 1 |  |  |  |  |  |  |  |  |  |
| 21 Ravens Matrices | **-.087** | **-.132** | **.452** | **.241** | **.335** | **.299** | -.027 | .018 | **.138** | -.028 | .021 | -.038 | -.008 | .041 | -.005 | -.080 | **.270** | **.236** | **-.212** | **.709** | 1 |  |  |  |  |  |  |  |  |
| 22 Verbal Fluency | -.036 | .037 | **.331** | **.162** | **.250** | **.175** | .019 | **.116** | **.155** | **-.123** | -.084 | **-.100** | .003 | -.072 | -.051 | -.019 | **.105** | .045 | **-.153** | **.408** | **.278** | 1 |  |  |  |  |  |  |  |
| 23 Logical Memory | -.023 | -.043 | **.264** | **.182** | **.213** | **.188** | .007 | **.092** | .090 | -.004 | -.042 | -.062 | .001 | -.025 | -.023 | **-.166** | .071 | **-.111** | -.082 | **.451** | **.388** | **.176** | 1 |  |  |  |  |  |  |
| 24 NART | **-.104** | .002 | **.670** | **.334** | **.538** | **.497** | -.036 | **.103** | **.148** | **-.111** | -.078 | -.032 | -.036 | .032 | .010 | -.073 | **.199** | **.111** | **-.138** | **.665** | **.461** | **.472** | **.319** | 1 |  |  |  |  |  |
| 25 HADS | .012 | **.096** | -.078 |  | **-.119** | **-.091** | -.018 | -.073 | **-.241** | **-.055** | **.109** | -.001 | **.091** | .016 | .035 | -.007 | **-.136** | **-.157** | **.203** | **-.156** | **-.163** | **-110** | **-.085** | **-114** | 1 |  |  |  |  |
| 26 WHOQOL | -.057 | -.080 | .078 | -.054 | **.158** | **.226** | -.061 | -.022 | **.446** | **-.094** | **-.123** | -.026 | -.077 | -.083 | -.075 | .005 | **.196** | **.175** | **-.379** | **.239** | **.202** | **.157** | **.122** | **.203** | **-.519** | 1 |  |  |  |
| 27 SWLS | -.058 | -.052 | .012 | -.040 | **.096** | **.139** | -.046 | -.006 | **.286** | -.027 | -.018 | -.030 | -.053 | -.024 | -.026 | -.014 | **.099** | .075 | **-.171** | .060 | **.092** | .046 | **.100** | .065 | **-.371** | **.652** | 1 |  |  |
| 28 Emotional stability | .016 | -.051 | **.106** | **.138** | **.183** | **.095** | -.001 | -.013 | **.235** | **.102** | -.022 | -.004 | -.023 | -.058 | -.047 | -.054 | **.093** | **.151** | **-.204** | **.208** | **.146** | **.126** | **.118** | **.137** | **-.483** | **.450** | **.265** | 1 |  |
| 29 Conscientiousness | -.017 | .010 | .002 | -.007 | -.071 | -.064 | -.059 | **-.131** | **.124** | .037 | .005 | -.012 | .014 | -.047 | -.020 | .015 | -.050 | .018 | -.066 | .004 | .048 | .012 | -.010 | -.089 | **-.227** | **.248** | **.172** | **.187** | 1 |

CVD, cardiovascular disease; FEV1, forced expiratory volume in 1 second; NART, National Adult Reading Test; HADS, Hospital Anxiety and Depression Scale; WHOQOL, World Health Organisation Quality of Life; SWLS, Satisfaction with Life Scale

P < 0.001

P < 0.01

P < 0.05

Note: Pearson r-values highlighted in colour according to significance level:

**eTable 2.** Variable information and missing data at baseline

| **Predictor variable** | **Description** | **Type** | **Coding/possible scoring range** | **Direction of coding**  **i.e., higher value =** | **Missing N (% of full sample if >10%)** |
| --- | --- | --- | --- | --- | --- |
| Age | Age in days at baseline | Continuous | Age in days | Older age | 0 |
| Sex | Sex | Binary | Male (0) or female (1) | Female | 0 |
| Childhood social class | Father’s occupational social class | Binary | Prof/managerial (1) or partly-skilled/unskilled (2) | Less professional class | 81 (15%) |
| Age 11 IQ | Moray House Test (MHT) score from age 11 | Continuous | Raw MHT score converted to an IQ-type score (mean = 100, sd = 15) | Higher IQ | 56 (10%) |
| Education | Number of years of formal full-time education | Continuous | Education in years | More education | 2 |
| Mid-life social class | Own occupational social class in adulthood | Binary | Prof/managerial (1) or partly-skilled/unskilled (2) | Less professional class | 2 |
| Smoking | Smoking status | Categorical | Never (0) former (1) current (2) | More smoking | 1 |
| Alcohol | Alcohol consumption, units per week | Continuous | Alcohol units per week | Higher alcohol consumption | 0 |
| Physical activity | Leisure activity questionnaire – total score | Continuous | Test score range (0 – 39) | More physical activity | 82 (15%) |
| Body mass index | Body mass index measured by nurse | Continuous | Body mass index score | Higher body mass index | 6 |
| CVD | History of cardiovascular disease | Binary | No (0) or yes (1) | History of disease | 5 |
| Stroke | History of stroke | Binary | No (0) or yes (1) | History of disease | - |
| Cancer | History of cancer | Binary | No (0) or yes (1) | History of disease | - |
| Hypertension | History of hypertension | Binary | No (0) or yes (1) | History of disease | 2 |
| Diabetes | History of diabetes | Binary | No (0) or yes (1) | History of disease | - |
| *APOE* e4 | *APOE* e4 status | Binary | -ve (0) or +ve (1) | Positive *APOE* e4 status | 7 |
| FEV1 | Forced expiratory volume in 1 second | Continuous | FEV1 measure | Better lung function | 6 |
| Grip strength | Dynamometer grip strength in kilos | Continuous | Grip strength measure | Better grip strength | 6 |
| Six metre walk | Time taken to walk six metres in seconds | Continuous | Six metre walk measure | Slower walking speed | 9 |
| Age 79 IQ | Moray House Test (MHT) score from age 79 | Continuous | Raw MHT score converted to an IQ-type score (mean = 100, sd = 15) | Higher IQ | 10 |
| Raven’s Matrices | Psychometric test of reasoning | Continuous | Test score range (0 – 60) | Better cognitive ability | 7 |
| Logical Memory | Psychometric test of verbal memory | Continuous | Test score range (0 – 75) | Better cognitive ability | 2 |
| Verbal Fluency | Psychometric test of executive function | Continuous | No maximum score | Better cognitive ability | 4 |
| NART | National Adult Reading Test | Continuous | Test score range (0 – 50) | Better cognitive ability | 2 |
| Emotional health | Hospital Anxiety and Depression Scale (HADS | Continuous | Test score range (0 – 42) | More anxiety/depressive symptoms | 3 |
| Quality of life | WHOQOL-BREF Quality of Life Scale | Continuous | Test score range (0 – 80) | Higher QOL | 77 (14%) |
| Life satisfaction | Satisfaction with Life Scale (SWLS) | Continuous | Test score range (5 – 35) | Higher life satisfaction | 81 (15%) |
| Emotional stability | International Personality Item Pool (IPIP) trait score | Continuous | Test score range (0 – 40) | Higher emotional stability | 100 (18%) |
| Conscientiousness | International Personality Item Pool (IPIP) trait score | Continuous | Test score range (0 – 40) | Higher conscientiousness | 100 (18%) |

CVD, cardiovascular disease; FEV1, forced expiratory volume in 1 second; NART, National Adult Reading Test; HADS, Hospital Anxiety and Depression Scale; WHOQOL, World Health Organisation Quality of Life

**eTable 3.** Characteristics of the sample according to full vs incomplete data

|  | **Full sample** | **Full data** | **Incomplete data** |  |
| --- | --- | --- | --- | --- |
|  | M (SD) or N (%) | M (SD) or N (%) | M (SD) or (N %) | P |
| N | 547 (100%) | 341 (62.3%) | 206 (37.8%) |  |
| Age (years) | 79.07 (0.6) | 79.09 (0.6) | 79.05 (0.6) | 0.539 |
| Sex, female | 547 (57.6%) | 198 (58.0%) | 117 (56.8%) | 0.771 |
| Survival in years | 89.50 (5.3) | 90.39 (5.0) | 88.01 (5.5) | **<0.001** |
| ***Early-life*** |  |  |  |  |
| Childhood social class |  |  |  | 0.424 |
| I (professional) | 46 (8.4%) | 30 (8.8%) | 16 (7.8%) |  |
| II | 122 (22.3%) | 94 (27.6%) | 28 (13.6%) |  |
| III | 228 (41.7%) | 169 (49.6%) | 59 (28.6%) |  |
| IV | 50 (9.1%) | 33 (9.7%) | 17 (8.3%) |  |
| V (unskilled) | 20 (3.7%) | 15 (4.4%) | 5 (2.4%) |  |
| Age 11 IQ | 100.00 (15.0) | 101.14 (14.2) | 97.35 (6.3) | **0.010** |
| Education (years) | 10.92 (2.5) | 10.98 (2.4) | 10.81 (2.5) | 0.432 |
|  |  |  |  |  |
| ***Mid-life*** |  |  |  |  |
| Mid-life social class |  |  |  | 0.113 |
| I (professional) | 128 (23.4%) | 75 (22.0%) | 53 (25.7%) |  |
| II | 181 (33.1%) | 126 (37.0%) | 55 (26.7%) |  |
| III | 217 (39.7%) | 131 (38.4%) | 86 (41.7%) |  |
| IV | 12 (2.2%) | 5 (1.5%) | 7 (3.4%) |  |
| V (unskilled) | 7 (1.3%) | 4 (1.2%) | 3 (1.5%) |  |
|  |  |  |  |  |
| ***Later-life*** |  |  |  |  |
| Smoking |  |  |  | **0.004** |
| Never | 235 (43.0%) | 161 (47.2%) | 74 (35.9%) |  |
| Former | 271 (49.5%) | 163 (47.8%) | 108 (52.4%) |  |
| Current | 40 (7.3%) | 17 (5.0%) | 23 (11.2%) |  |
| Alcohol intake (units) | 5.73 (10.7) | 4.96 (8.3) | 7.00 (13.8)) | **0.031** |
| Physical activity | 22.30 (5.7) | 22.71 (5.7) | 21.18 (5.5) | **0.010** |
| Body mass index | 26.23 (4.2) | 26.14 (4.1) | 26.40 (4.2) | 0.480 |
| CVD: % yes | 163 (29.8%) | 105 (30.8%) | 58 (28.2%) | 0.354 |
| Stroke: % yes | 46 (8.4%) | 32 (9.4%) | 14 (6.8%) | 0.185 |
| Cancer: % yes | 54 (9.9%) | 31 (9.1%) | 23 (11.2%) | 0.259 |
| Hypertension: % yes | 219 (40.0%) | 147 (43.1%) | 72 (35.0%) | **0.043** |
| Diabetes: % yes | 28 (5.1%) | 12 (3.5%) | 16 (7.8%) | **0.025** |
| *APOE* e4 carrier: % yes | 145 (26.5%) | 85 (24.9%) | 60 (29.1%) | 0.186 |
| FEV in 1 sec | 1.88 (0.6) | 1.92 (0.6) | 1.81 (0.6) | 0.053 |
| Grip strength | 26.54 (9.1) | 26.63 (9.1) | 26.33 (9.1) | 0.710 |
| Six metre walk (secs) | 4.74 (1.9) | 4.59 (1.7) | 5.00 (2.3) | **0.018** |
| Moray House Test score | 59.23 (10.8) | 60.51 (10.6) | 58.29 (10.9) | **<0.001** |
| Age 79 IQ | 100 (15.0) | 102.16 (12.9) | 96.22 (17.5) | **<0.001** |
| Ravens Matrices score | 31.16 (8.8) | 32.06 (8.0) | 29.58 (9.9) | **0.002** |
| Verbal Fluency | 39.94 (12.3) | 41.17 (11.9) | 37.87 (12.6) | **0.002** |
| Logical Memory | 31.64 (12.8) | 32.24 (12.8) | 30.64 (12.7) | 0.158 |
| NART | 34.17 (8.3) | 34.90 (7.9) | 32.96 (8.8) | **0.008** |
| Emotional health (HADS) | 8.76 (4.7) | 8.47 (4.7) | 9.25 (4.8) | 0.060 |
| Quality of life (WHOQOL) | 63.91 (7.3) | 64.34 (7.0) | 62.61 (8.0) | **0.022** |
| Life satisfaction (SWLS) | 25.34 (6.1) | 25.56 (6.0) | 24.63 (6.2) | 0.142 |
| Emotional stability | 24.34 (8.1) | 24.64 (7.8) | 23.28 (9.0) | 0.133 |
| Conscientiousness | 28.71 (6.1) | 28.98 (5.9) | 27.81 (6.4) | 0.083 |

CVD, cardiovascular disease; FEV1, forced expiratory volume in 1 second; NART, National Adult Reading Test; HADS, Hospital Anxiety and Depression Scale; WHOQOL, World Health Organisation Quality of Life; SWLS, Satisfaction with Life Scale

Boldtype indicates statistical significance, P < 0.05

**eTable 4.** Sensitivity analysis: Multivariable Cox proportional hazards model predicting mortality with an earlier census date (21 March 2013)

|  | **Multivariable model**  **(all covariates mutually-adjusted)** | |
| --- | --- | --- |
|  | **Hazard ratios (95% CI)** | **P-value** |
| Age (in days) | 0.93 (0.80, 1.07) | 0.297 |
| Sex (ref: males) | 0.85 (0.60, 1.19) | 0.343 |
| **Early-life** |  |  |
| Social class (ref: prof/managerial) |  |  |
| Partly skilled/unskilled | 1.48 (1.05, 2.07) | ɫ0.024 |
| Age 11 IQ score | 1.18 (0.95, 1.46) | 0.133 |
| Education (years) | 1.00 (0.93, 1.08) | 0.992 |
| **Mid-life** |  |  |
| Social class (ref: prof/managerial) |  |  |
| Partly skilled/unskilled | 1.08 (0.78, 1.49) | 0.638 |
| **Later-life: lifestyle** |  |  |
| Smoking (ref: never) |  |  |
| Former smoking | 1.14 (0.83, 1.54) | 0.420 |
| Current smoking | 1.00 (0.51, 1.96) | 0.994 |
| Alcohol intake (units) | 1.23 (1.01, 1.50) | ɫ0.037 |
| Physical activity score | 0.78 (0.66, 0.92) | **0.003** |
| Body mass index | 0.94 (0.82, 1.09) | 0.430 |
| **Later-life: physical health** |  |  |
| CVD (ref: no history) | 1.15 (0.83, 1.59) | 0.411 |
| Stroke (ref: no history) | 0.75 (0.45, 1.25) | 0.270 |
| Cancer (ref: no history) | 1.81 (1.15, 2.85) | **0.010** |
| Hypertension (ref: no history) | 1.12 (0.84, 1.51) | 0.443 |
| Diabetes (ref: no history) | 2.13 (1.07, 4.26) | ɫ0.032 |
| **Later-life: functional health** |  |  |
| Physical function (latent factor) | 0.52 (0.35, 0.77) | **0.001** |
| FEV in 1 sec |  |  |
| Grip strength |  |  |
| Six metre walk (secs) |  |  |
| **Later-life: cognitive health** |  |  |
| Cognitive function (latent factor) | 0.73 (0.56, 0.94) | **0.015** |
| Age 79 IQ score |  |  |
| Ravens Matrices score |  |  |
| Verbal Fluency score |  |  |
| Logical Memory score |  |  |
| NART score |  |  |
| **Later-life: psychosocial health** |  |  |
| Emotional health (HADS) | 1.01 (0.86, 1.20) | 0.880 |
| Quality of life (WHOQOL) | 1.19 (0.95, 1.50) | 0.134 |
| Satisfaction with life (SWLS) | 0.96 (0.80, 1.15) | 0.626 |
| **Intrinsic: genetic** |  |  |
| *APOE* e4 status (ref: -ve) | 1.08 (0.78, 1.49) | 0.651 |
| **Intrinsic: personality** |  |  |
| Emotional stability | 0.98 (0.82, 1.17) | 0.799 |
| Conscientiousness | 0.99 (0.86, 1.15) | 0.941 |

Note. CVD, cardiovascular disease; FEV1, forced expiratory volume in 1 second; NART, National Adult Reading Test; HADS, Hospital Anxiety and Depression Scale; WHOQOL, World Health Organisation Quality of Life; SWLS, Satisfaction with Life Scale.

Survival time was calculated as either the number of days between date of birth and date of death or right census date.

Physical function is a latent variable derived from FEV1, grip strength and six-metre walk measures. Cognitive function is a latent variable derived from scores on the Moray House Test, Raven’s Matrices, Verbal Fluency, Logical Memory and the NART.

Unless indicated with a corresponding reference category, measures were continuous.

We examined all measures simultaneously in one single model.

ɫindicates statistical significance at the P < 0.05 level, before FDR correction.

Boldtype indicates P values which survive FDR adjustment.

**eTable 5.** Structural life-course model predicting survival: includes direct paths to survival from all covariates, and additional paths among early-life variables, and from early-life and intrinsic (stable) variables to later-life variables

|  | **Survival** | | | | |
| --- | --- | --- | --- | --- | --- |
|  | **Std est** | **95% CI** | **z** | **HR*** | **P-value** |
| ***Direct paths to survival*** |  |  |  |  |  |
| Age | .013 | -0.07, 0.09 | 0.327 | 1.01 | 0.744 |
| Sex | .139 | 0.05, 0.23 | 3.059 | 1.32 | **0.002** |
| **Early-life** |  |  |  |  |  |
| Childhood social class | .037 | -0.13, 0.06 | 0.765 | 1.04 | 0.444 |
| Age 11 IQ | -.130 | -0.25, -0.01 | -2.096 | 0.88 | ɫ0.036 |
| Education | -.014 | -0.12, 0.09 | -0.278 | 0.99 | 0.781 |
| **Mid-life** |  |  |  |  |  |
| Mid-life social class | .023 | -0.13, 0.07 | -0.463 | 1.04 | 0.643 |
| **Later-life: lifestyle** |  |  |  |  |  |
| Smoking category | -.115 | -0.20, -0.03 | -2.780 | 0.83 | **0.005** |
| Alcohol units | -.011 | -0.09, 0.07 | -0.251 | 0.99 | 0.801 |
| Physical activity | .150 | 0.05, 0.25 | 3.058 | 1.16 | **0.002** |
| Body mass index | .004 | -0.08, 0.09 | 0.094 | 1.00 | 0.925 |
| **Later-life: physical health** |  |  |  |  |  |
| CVD | -.048 | -0.13, 0.03 | -1.156 | 0.90 | 0.248 |
| Stroke | .065 | -0.01, 0.14 | 1.599 | 1.26 | 0.110 |
| Cancer | -.132 | -0.21, -0.05 | -3.283 | 0.65 | **0.001** |
| Hypertension | -.085 | -0.16, -0.01 | -2.070 | 0.84 | ɫ0.038 |
| Diabetes | -.091 | -0.17, -0.01 | -2.242 | 0.67 | ɫ0.025 |
| **Later-life: functional health** |  |  |  |  |  |
| Physical function (latent) | .184 | 0.10, 0.27 | 4.274 | 1.58 | **<0.001** |
| **Later-life: cognitive health** |  |  |  |  |  |
| Cognitive function (latent) | .204 | 0.09, 0.32 | 3.369 | 1.24 | **<0.001** |
| **Later-life: psychosocial** |  |  |  |  |  |
| Emotional health (HADS) | -.009 | -0.11, 0.09 | -0.176 | 0.99 | 0.860 |
| Quality of life (WHOQOL) | -.020 | -0.16, 0.11 | -0.298 | 0.98 | 0.766 |
| Satisfaction with life (SWLS) | -.028 | -0.14, 0.08 | -0.499 | 0.97 | 0.618 |
| **Intrinsic: genetic** |  |  |  |  |  |
| *APOE* e4 status | -.055 | -0.13, 0.03 | -1.345 | 0.89 | 0.178 |
| **Intrinsic: personality** |  |  |  |  |  |
| Emotional stability | -.004 | -0.11, 0.10 | -0.082 | 0.10 | 0.935 |
| Conscientiousness | .041 | -0.05, 0.14 | 0.861 | 1.04 | 0.389 |
|  |  |  |  |  |  |
| ***Paths between early-life variables*** |  |  |  |  |  |
| Childhood social class → Age 11 IQ | .299 | -0.37, -0.19 | 6.057 | - | **<0.001** |
| Childhood social class → Education | .861 | -0.40, -0.25 | 8.139 | - | **<0.001** |
| Childhood social class → Mid-life social class | .077 | -0.01, 0.17 | 1.836 | - | 0.066 |
| Age 11 IQ → Education | .887 | 0.29, 0.44 | 9.231 | - | **<0.001** |
| Age 11 IQ → Mid-life social class | .221 | -0.33, -0.17 | 5.981 | - | **<0.001** |
| Education → Mid-life social class | .118 | -0.41, -0.25 | 7.477 | - | **<0.001** |
|  |  |  |  |  |  |
| ***Indirect paths from early- to later-life variables*** |  |  |  | - |  |
| Childhood social class → Smoking | 0.105 | -0.20, -0.01 | 2.070 | - | ɫ0.038 |
| Childhood social class → Alcohol | 0.082 | -0.18, 0.01 | 1.668 | - | 0.095 |
| Childhood social class → Activity | 0.036 | -0.13, 0.06 | 0.720 | - | 0.472 |
| Childhood social class → BMI | -0.006 | -0.09, 0.11 | -0.127 | - | 0.899 |
| Childhood social class → CVD | 0.108 | -0.21, -0.01 | 2.116 | - | ɫ0.034 |
| Childhood social class → Stroke | -0.055 | -0.04, 0.15 | -1.097 | - | 0.273 |
| Childhood social class → Cancer | 0.002 | -0.10, 0.10 | 0.036 | - | 0.971 |
| Childhood social class → Hypertension | -0.026 | -0.08, 0.13 | -0.503 | - | 0.615 |
| Childhood social class → Diabetes | 0.042 | -0.14, 0.06 | 0.821 | - | 0.411 |
| Childhood social class → Physical function | -0.047 | -0.05, 0.14 | -0.959 | - | 0.338 |
| Childhood social class → Cognitive function | 0.078 | -0.15, -0.00 | 2.019 | - | ɫ0.043 |
| Childhood social class → Emotional health | -0.010 | -0.08, 0.10 | -0.207 | - | 0.836 |
| Childhood social class → Quality of life | -0.080 | 0.01, 0.17 | -1.797 | - | 0.072 |
| Childhood social class → Satisfaction with life | -0.037 | -0.06, 0.13 | -0.735 | - | 0.462 |
|  |  |  |  |  |  |
| Age 11 IQ → Smoking | 0.013 | -0.08, 0.11 | 0.255 | - | 0.799 |
| Age 11 IQ → Alcohol | -0.005 | -0.11, 0.10 | -0.106 | - | 0.916 |
| Age 11 IQ → Activity | -0.074 | -0.18, 0.03 | -1.378 | - | 0.168 |
| Age 11 IQ → BMI | -0.049 | -0.15, 0.05 | -0.933 | - | 0.351 |
| Age 11 IQ → CVD | -0.039 | -0.14, 0.06 | -0.773 | - | 0.439 |
| Age 11 IQ → Stroke | 0.009 | -0.09, 0.11 | 0.173 | - | 0.862 |
| Age 11 IQ → Cancer | 0.022 | -0.08, 0.12 | 0.426 | - | 0.670 |
| Age 11 IQ → Hypertension | 0.023 | -0.08, 0.12 | 0.439 | - | 0.661 |
| Age 11 IQ → Diabetes | 0.006 | -0.10, 0.11 | 0.118 | - | 0.906 |
| Age 11 IQ → Physical function | -0.019 | -0.11, 0.08 | -0.390 | - | 0.697 |
| Age 11 IQ → Cognitive function | 0.579 | 0.52, 0.64 | 16.075 | - | **<0.001** |
| Age 11 IQ → Emotional health | -0.024 | -0.11, 0.07 | -0.530 | - | 0.596 |
| Age 11 IQ → Quality of life | -0.059 | -0.16, 0.04 | -1.171 | - | 0.241 |
| Age 11 IQ → Satisfaction with life | -0.097 | -0.21, 0.01 | -1.738 | - | 0.082 |
|  |  |  |  |  |  |
| Education → Smoking | -0.222 | -0.33, -0.12 | -4.156 | - | **<0.001** |
| Education → Alcohol | -0.013 | -0.12, 0.09 | -0.240 | - | 0.810 |
| Education → Activity | 0.093 | -0.02, 0.20 | 1.676 | - | 0.094 |
| Education → BMI | 0.003 | -0.11, 0.11 | 0.054 | - | 0.957 |
| Education → CVD | -0.077 | -0.18, 0.03 | -1.419 | - | 0.156 |
| Education → Stroke | -0.005 | 0.11, 0.10 | -0.084 | - | 0.933 |
| Education → Cancer | -0.020 | -0.13, 0.09 | -0.360 | - | 0.719 |
| Education → Hypertension | -0.049 | -0.16, 0.06 | -0.890 | - | 0.373 |
| Education → Diabetes | 0.005 | -0.10, 0.11 | 0.088 | - | 0.930 |
| Education → Physical function | 0.093 | -0.01, 0.20 | 1.796 | - | 0.073 |
| Education → Cognitive function | 0.096 | 0.02, 0.17 | 2.418 | - | **0.016** |
| Education → Emotional health | -0.024 | -0.12, 0.07 | -0.495 | - | 0.620 |
| Education → Quality of life | 0.064 | -0.04, 0.16 | 1.261 | - | 0.207 |
| Education → Satisfaction with life | 0.056 | -0.05, 0.17 | 0.993 | - | 0.321 |
|  |  |  |  |  |  |
| Mid-life social class → Smoking | 0.007 | -0.11, 0.09 | 0.149 | - | 0.881 |
| Mid-life social class → Alcohol | 0.109 | -0.21, -0.01 | 2.236 | - | ɫ0.025 |
| Mid-life social class → Activity | 0.176 | -0.28, -0.08 | 3.375 | - | **0.001** |
| Mid-life social class → BMI | -0.057 | -0.05, 0.16 | -1.095 | - | 0.274 |
| Mid-life social class → CVD | -0.009 | -0.09, 0.11 | -0.183 | - | 0.855 |
| Mid-life social class → Stroke | 0.018 | -0.12, 0.08 | 0.359 | - | 0.720 |
| Mid-life social class → Cancer | -0.023 | -0.08, 0.12 | -0.452 | - | 0.651 |
| Mid-life social class → Hypertension | 0.027 | -0.13, 0.07 | 0.521 | - | 0.603 |
| Mid-life social class → Diabetes | -0.023 | -0.08, 0.12 | -0.449 | - | 0.653 |
| Mid-life social class → Physical function | 0.023 | -0.12, 0.07 | 0.462 | - | 0.644 |
| Mid-life social class → Cognitive function | 0.068 | -0.14, 0.00 | 1.889 | - | ɫ0.059 |
| Mid-life social class → Emotional health | -0.026 | -0.06, 0.12 | -0.569 | - | 0.569 |
| Mid-life social class → Quality of life | 0.207 | -0.30, -0.12 | 4.351 | - | **<0.001** |
| Mid-life social class → Satisfaction with life | 0.150 | -0.25, -0.05 | 2.856 | - | **0.004** |
|  |  |  |  |  |  |
| ***Indirect paths from intrinsic- to later-life variables*** |  |  |  |  |  |
| Age → Smoking | -0.015 | -0.10, 0.07 | -0.350 | - | 0.727 |
| Age → Alcohol | 0.006 | -0.07, 0.09 | 0.144 | - | 0.885 |
| Age → Activity | -0.011 | -0.10, 0.07 | -0.254 | - | 0.800 |
| Age → BMI | -0.004 | -0.09, 0.08 | -0.089 | - | 0.929 |
| Age → CVD | 0.032 | -0.05, 0.12 | 0.765 | - | 0.444 |
| Age → Stroke | -0.010 | -0.09, 0.07 | -0.231 | - | 0.818 |
| Age → Cancer | -0.043 | -0.13, 0.04 | -0.995 | - | 0.320 |
| Age → Hypertension | 0.010 | -0.07, 0.09 | 0.225 | - | 0.822 |
| Age → Diabetes | 0.029 | -0.06, 0.11 | 0.667 | - | 0.504 |
| Age → Physical function | -0.036 | -0.12, 0.04 | -0.882 | - | 0.378 |
| Age → Cognitive function | -0.002 | -0.06, 0.06 | -0.073 | - | 0.942 |
| Age → Emotional health | 0.013 | -0.06, 0.09 | 0.340 | - | 0.734 |
| Age → Quality of life | -0.041 | -0.12, 0.04 | -1.054 | - | 0.292 |
| Age → Satisfaction with life | -0.052 | -0.14, 0.03 | -1.206 | - | 0.228 |
|  |  |  |  |  |  |
| Sex → Smoking | -0.129 | -0.21, -0.05 | -3.064 | - | **0.002** |
| Sex → Alcohol | -0.235 | -0.31, -0.16 | -5.690 | - | **<0.001** |
| Sex → Activity | 0.142 | 0.06, 0.23 | 3.255 | - | **0.001** |
| Sex → BMI | 0.004 | -0.08, 0.09 | 0.101 | - | 0.920 |
| Sex → CVD | -0.171 | -0.25, -0.09 | -4.023 | - | **<0.001** |
| Sex → Stroke | -0.064 | -0.15, 0.02 | -1.476 | - | 0.140 |
| Sex → Cancer | -0.059 | -0.14, 0.03 | -1.375 | - | 0.169 |
| Sex → Hypertension | 0.054 | -0.03, 0.14 | 1.246 | - | 0.213 |
| Sex → Diabetes | -0.021 | -0.11, 0.06 | -0.487 | - | 0.626 |
| Sex → Physical function | -0.298 | -0.37, -0.22 | -7.241 | - | **<0.001** |
| Sex → Cognitive function | -0.062 | -0.12, -0.00 | -2.020 | - | ɫ0.043 |
| Sex → Emotional health | 0.066 | -0.01, 0.14 | 1.725 | - | 0.085 |
| Sex → Quality of life | -0.036 | -0.11, 0.04 | -0.918 | - | 0.359 |
| Sex → Satisfaction with life | -0.019 | -0.11, 0.07 | -0.436 | - | 0.663 |
|  |  |  |  |  |  |
| Emotional stability → Smoking | 0.023 | -0.07, 0.11 | 0.494 | - | 0.621 |
| Emotional stability → Alcohol | -0.020 | -0.12, 0.08 | -0.414 | - | 0.679 |
| Emotional stability → Activity | 0.220 | 0.13, 0.31 | 4.767 | - | **<0.001** |
| Emotional stability → BMI | 0.085 | -0.01, 0.18 | 1.784 | - | 0.074 |
| Emotional stability → CVD | -0.026 | -0.12, 0.07 | -0.551 | - | 0.581 |
| Emotional stability → Stroke | -0.016 | -0.11, 0.08 | -0.343 | - | 0.732 |
| Emotional stability → Cancer | -0.048 | -0.14, 0.05 | -0.991 | - | 0.322 |
| Emotional stability → Hypertension | -0.018 | -0.11, 0.07 | -0.387 | - | 0.699 |
| Emotional stability → Diabetes | -0.060 | -0.16, 0.04 | -1.223 | - | 0.222 |
| Emotional stability → Physical function | 0.121 | 0.03, 0.21 | 2.639 | - | **0.008** |
| Emotional stability → Cognitive function | 0.132 | 0.06, 0.20 | 3.800 | - | **<0.001** |
| Emotional stability → Emotional health | -0.441 | -0.51, -0.37 | -10.688 | - | **<0.001** |
| Emotional stability → Quality of life | 0.424 | 0.35, 0.50 | 10.050 | - | **<0.001** |
| Emotional stability → Satisfaction with life | 0.249 | 0.16, 0.34 | 5.396 | - | **<0.001** |
|  |  |  |  |  |  |
| Conscientiousness → Smoking | -0.095 | -0.19, -0.00 | -2.023 | - | ɫ0.043 |
| Conscientiousness → Alcohol | -0.141 | -0.24, -0.04 | -2.794 | - | **0.005** |
| Conscientiousness → Activity | 0.120 | 0.03, 0.21 | 2.577 | - | **0.010** |
| Conscientiousness → BMI | -0.005 | -0.10, 0.09 | -0.095 | - | 0.924 |
| Conscientiousness → CVD | 0.004 | -0.09, 0.10 | 0.078 | - | 0.938 |
| Conscientiousness → Stroke | -0.016 | -0.11, 0.08 | -0.339 | - | 0.735 |
| Conscientiousness → Cancer | 0.009 | -0.09, 0.11 | 0.187 | - | 0.852 |
| Conscientiousness → Hypertension | -0.031 | -0.13, 0.06 | -0.649 | - | 0.517 |
| Conscientiousness → Diabetes | -0.018 | -0.12, 0.08 | -0.360 | - | 0.719 |
| Conscientiousness → Physical function | 0.035 | -0.06, 0.13 | 0.747 | - | 0.455 |
| Conscientiousness → Cognitive function | 0.048 | -0.02, 0.12 | 1.350 | - | 0.177 |
| Conscientiousness → Emotional health | -0.146 | -0.23, -0.07 | -3.489 | - | **<0.001** |
| Conscientiousness → Quality of life | 0.202 | 0.12, 0.28 | 4.769 | - | **<0.001** |
| Conscientiousness → Satisfaction with life | 0.147 | 0.06, 0.24 | 3.163 | - | **0.002** |

BMI, body mass index; CVD, cardiovascular disease

Survival time was calculated as the number of days between date of birth and date of death, or date of birth and right census date

*Hazard ratios for the direct effects are exponentiated beta values

Physical function is a latent variable derived from FEV1, grip strength and six-metre walk; Cognitive function is a latent variable derived from scores on the Moray House Test, Raven’s Matrices, Verbal Fluency, Logical Memory and NART

^ɫ^indicates statistical significance, where P < 0.05, before FDR correction

Boldtype indicates P values which survive FDR adjustment
